# Questionnaire-Based Survey Regarding the Rational Usage of Antimicrobial Agents in Food-Producing Animals in Romania

**DOI:** 10.3390/vetsci12050408

**Published:** 2025-04-26

**Authors:** Ionela Popa, Kalman Imre, Adriana Morar, Ionica Iancu, Vlad Iorgoni, Timea Bochiș, Călin Pop, Alexandru Gligor, Tiana Florea, Sebastian Alexandru Popa, Viorel Herman, Ileana Nichita

**Affiliations:** Faculty of Veterinary Medicine, University of Life Sciences “King Mihai I” from Timişoara, 300645 Timișoara, Romania; adrianamorar@usvt.ro (A.M.); ionica.iancu@usvt.ro (I.I.); vlad.iorgoni@usvt.ro (V.I.); timea.bochis@usvt.ro (T.B.); calinpop@usvt.ro (C.P.); alexandru.gligor@usvt.ro (A.G.); tijana.florea@usvt.ro (T.F.); sebastian.popa@usvt.ro (S.A.P.); viorel.herman@fmvt.ro (V.H.); ileananichita@usvt.ro (I.N.)

**Keywords:** questionnaire, antimicrobial resistance, concern, food-producing animals

## Abstract

The annual rise in antimicrobial resistance (AMR) is a major public health concern. This Romanian questionnaire-based survey aimed to evaluate the appropriateness of routine antimicrobial use by veterinary practitioners in food-producing animals and to gauge their concerns about AMR. To gather data, an online questionnaire with 28 questions was developed and distributed nationwide to veterinarians via the Google Drive network and Google Forms. Despite this increased awareness, deviations in antimicrobial administration persist, often due to insufficient knowledge about AMR and the mechanisms of antimicrobial action. This study underscores the urgent need for prudent antimicrobial use in livestock production in Romania.

## Simple Summary

This study addresses the critical issue of antimicrobial resistance (AMR) in Romania, a growing public health threat. Through a questionnaire-based survey, this study evaluated how veterinarians use antimicrobials in food-producing animals and assessed their concerns about AMR. Data were collected from 80 veterinarians nationwide. The results show that while the awareness of AMR risks is increasing, deviations in antimicrobial use still occur, often due to a lack of sufficient knowledge about AMR and how these drugs work. The findings underscore the urgent need for responsible antimicrobial stewardship in livestock medicine. Specifically, Romanian veterinarians would benefit from more precise guidelines on antimicrobial use, particularly in surgical procedures such as C-sections. Clearer protocols on when antimicrobials are warranted and which agents to use would help optimize therapeutic outcomes while mitigating antimicrobial resistance risks.

## 1. Introduction

Antimicrobial resistance (AMR) is a serious global health concern, one of the greatest of this century [1,2,3,4,5,6]. Currently, it is responsible for the death of 700,000 people annually, but it is estimated that 10 million lives could be at risk annually by 2050. Additionally, AMR has not only a social impact but also an economic one, and if this trend continues, by 2050, it will lead to a total cost of USD 100 trillion, affecting the economy to the same degree as the financial crisis of 2008 [7,8].

Antimicrobial resistance (AMR) is a growing concern worldwide, and Romania is no exception, with increasing rates of resistance to commonly used antibiotics. A recent survey conducted among 2500 adults in Romania revealed significant gaps in knowledge, attitudes, and practices regarding antibiotic use and AMR. The study found that only a small percentage of respondents had accurate knowledge of antibiotic efficacy and resistance mechanisms, with many participants unaware of the risks associated with inappropriate antibiotic use. Notably, over half of the respondents had a negative attitude toward antibiotic consumption, with 52.73% of individuals agreeing that they always try to have an antibiotic at home, which is considered an improper practice. Furthermore, a considerable portion of the population engages in self-medication, using antibiotics without medical prescriptions. These behaviors contribute to the spread of AMR, which is exacerbated by the lack of public awareness about the severity of the issue. The survey highlighted that improper antibiotic use, especially in food-producing animals and through self-medication, is a major factor in the development and dissemination of AMR in Romania. As such, addressing these gaps in knowledge and behavior is essential for controlling the rise of AMR in the country [9].

Recent studies from the Banat region (Romania) have highlighted significant antimicrobial resistance (AMR) concerns in food products, particularly in dairy items. Escherichia coli isolates from raw milk cheese exhibited alarming resistance patterns, with complete (100%) resistance to enrofloxacin and notable resistance rates to ampicillin (39.5%) and norfloxacin (28.6%). Furthermore, 23.3% of these strains displayed multidrug resistance, underscoring serious public health implications [10,11].

Similarly, antimicrobial resistance has been observed in *Staphylococcus aureus* isolates from traditional cheeses in the region. These isolates showed the highest resistance to amikacin (90.1%), enrofloxacin (86.2%), and ceftiofur (72.7%). Multidrug resistance was detected in 49% of the strains, further highlighting the substantial risk to public health [10,11].

Antimicrobials are essential both for saving patients’ lives and for remarkable achievements in human and veterinary medicine and surgery [12]. Livestock production is essential for ensuring food security and generating economic benefits through the supply of animal products and income for farmers [13]. Antimicrobials are used in livestock production for therapeutic, metaphylactic, and prophylactic purposes, and in some countries, they continue to be used for growth promotion due to the improved feed conversion rates achieved after their administration [14].

Due to the administration of antimicrobials for the aforementioned purposes, AMR occurs not only in pathogenic bacteria but also in commensal bacteria [15,16].

AMR exemplifies the One Health approach, which integrates human, animal, and environmental health. The excessive use of antimicrobials in agriculture, medicine, and livestock promotes the development of resistance. Effective management of AMR requires a unified strategy that includes global awareness, improved hygiene, reduced antimicrobial use, and enhanced surveillance to address this complex and interconnected issue [16].

AMR bacteria, resistance genes, and antimicrobial residues are considered environmental pollutants and are primary contributors to the global public health crisis [15]. AMR bacteria result in limited treatment options in many developing countries, where the quality of treatment is often suboptimal, leading to infections becoming a major cause of mortality and morbidity [15,17].

AMR can spread from animals to humans due to the routine use of shared antimicrobials for bacterial infections of animal origin [15]. Zoonotic pathogens may develop resistance in livestock and transfer it to humans, promoting the dissemination of AMR genes across species.

The use of antimicrobials as growth promoters in livestock feed has been banned in the European Union since 2006, including in Romania [18,19]. This practice was first linked to antimicrobial resistance in the 1960s in the United Kingdom, leading to the Swann Committee’s recommendation to restrict antimicrobials important for human medicine [18]. The European Union progressively banned certain antimicrobial classes in 1999, followed by a complete ban in 2006 [18,19]. The United States implemented a similar restriction in 2017 [20].

The purpose of this study is closely related to the increasingly present and increasingly concerning threat posed by the emergence and continuous development of the phenomenon of AMR.

In this regard, a questionnaire prepared and addressed to veterinarians practicing on pigs and cattle aims to provide an overview of how correctly veterinarians use antimicrobials and to what extent they are concerned about the emergence of AMR. They were chosen because cattle and pigs receive high amounts of antibiotics, intensive farming increases the risk of resistant bacteria spreading, zoonotic transmission poses a public health threat, and regulatory monitoring is essential for optimizing antibiotic stewardship. Analyzing the 80 questionnaire responses helps assess practitioners’ antimicrobial use and identify potential mistakes. This study offers insights to improve antimicrobial practices in Romanian livestock, contributing to reduced use and antimicrobial resistance.

## 2. Materials and Methods

This study, which was approved by the Bioethics Commission of the University of Life Sciences “King Mihai I” from Timisoara No. 531/8 April 2025, utilized a structured questionnaire (see Supplementary Materials) to assess antimicrobial use among practicing veterinarians and identify areas for improvement in antimicrobial stewardship (AMS) to help mitigate antimicrobial resistance (AMR). The questionnaire was developed using a Google-based platform (Google Forms) and distributed at a national level via social media networks, including email, Facebook, and WhatsApp. The survey was available for completion from March to June 2022, targeting veterinarians working primarily with pigs and cattle, being sent only to veterinarians who were employed on pig or cattle farms.

The questionnaire was designed with a combination of dichotomous (yes/no), multiple-choice, open-ended, and rating scale questions to assess various aspects of antimicrobial usage. Open-ended questions collected demographic data, including participants’ age, antimicrobial selection criteria, the most commonly used route of antimicrobial administration, adherence to medication package inserts, the leaflet, the specific antimicrobials used for common cattle and swine diseases, and respondents’ reasoning for certain choices. Multiple-choice and dichotomous questions were used to examine antimicrobial administration practices and to assess the accuracy and compliance of veterinarians in following appropriate protocols. These questions also helped identify potential errors that could favor or accelerate the emergence of AMR.

A rating scale question was included to evaluate veterinarians’ level of concern regarding AMR. This aspect was considered critical, as heightened awareness of AMR could lead to more cautious antimicrobial use in veterinary practice.

To ensure accuracy in data analysis, responses were analyzed based on the number of valid answers provided for each question rather than the total number of participants. Some veterinarians did not respond to all questions, and the number of respondents for each item is specified in Section 3. This approach allowed for a more precise and reliable interpretation of the collected data.

The selection criteria for veterinarians were based on their field of activity, specifically those working with farm animals, particularly cattle and swine. The questionnaire was sent exclusively to veterinarians employed on pig and cattle farms or to district veterinarians whose practice predominantly involves pigs and cattle. It was not distributed to veterinarians working with other types of animals. All responses were collected anonymously.

The study included responses from 80 veterinarians; however, the number of answers varied for certain questions, as some respondents did not provide answers to all items. To ensure accurate data analysis, only the available responses were considered for each question. The reporting of results was performed relative to the number of responses obtained for each specific question, rather than the total number of participants. This approach allowed for a more precise representation of the collected data. The number of respondents for each question is explicitly mentioned in Section 3.

For responses that included the class of the antimicrobial, we assumed that they referred to the antimicrobial most frequently mentioned by other respondents. For responses where a product was mentioned, we recorded the antimicrobial and noted the product in parentheses; however, the presence of the product name in parentheses does not imply that the majority of veterinarians mentioned the product. Each was cited as an example, even if mentioned by only a few respondents, and in cases where a specific product was mentioned by the majority, this is specified.

The collected data (responses from the questionnaire) were collated using Microsoft Excel 2016. All questionnaire responses were automatically entered into this program, which was then used to create graphs and tables, which were created to facilitate better and easier descriptive analysis of the collected data.

In Romania, there are approximately 4000 veterinarians registered with the Romanian CMVRO (College of Veterinarians from Romania), of which over half (2350) are in rural areas [21] and less than half serve cattle and pig breeding units (approximately 1200); as a result, the number of respondents of the applied questionnaire (*n* = 80) would represent 6.6% of them.

## 3. Results

### 3.1. Information About Participants

The 80 veterinarians who completed the questionnaire ranged in age from 25 to 67 years and were categorized into four age groups. The majority were in the 25–35 age group (*n* = 64, 80%). The age distribution is illustrated in Table 1.

Most participants were male (73.8%, Table 1), with only 26.3% being female.

Experience in the field was another criterion studied. Slightly over half of the veterinarians participating in this study had less than five years of experience, with 54 individuals falling into this category (67.5%), making it the most numerous. Conversely, the category with 10–20 years of experience had the fewest participants, with only seven individuals (8.8%), as shown in Table 1.

### 3.2. General Aspects Regarding the Administration of Antibiotics

One of the most important aspects addressed in the questionnaire was to determine whether veterinarians perform antibiograms before using antimicrobials. When asked about the use of antibiograms, half of the veterinarian respondents answered negatively (*n* = 40, 50%), while the other half answered affirmatively (*n* = 40, 50%).

This study aimed to determine whether veterinarians are influenced by cost. Slightly over half of the respondents answered negatively regarding choosing a cheaper alternative antimicrobial. However, selecting a more affordable option is not inherently problematic. If an antibiogram indicates that two antimicrobials from the same risk category are effective, opting for the less expensive one is a reasonable choice. Additionally, narrow-spectrum antibiotics are often more cost-effective than broad-spectrum ones, aligning both antimicrobial stewardship (AMS) principles and cost considerations.

The question posed to veterinarians specifically asked whether, in cases where the most effective antibiotic indicated by the antibiogram has a high cost, they would choose or recommend a more affordable alternative with lower efficacy. The issue arises when a cheaper but less effective alternative is selected, as this can compromise treatment success and contribute to antimicrobial resistance.

**Figure 1:**
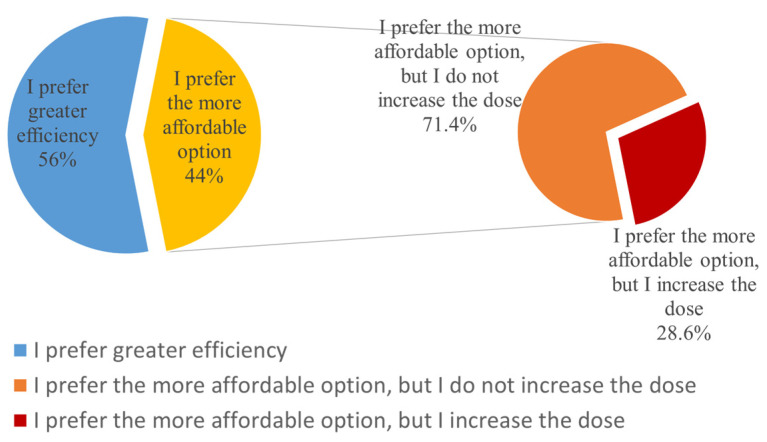
Influence of cost on the selection of antimicrobial agents by Romanian veterinarians. Highlighting how the cost of antimicrobials affects their prescribing decisions.

Specifically, 45 responses were negative (56%), while 35 veterinarians (44%) stated that they would choose a more affordable option. Among the 35 who indicated that they would choose a more affordable option, only 10 (28.6%) said that they would increase the dose, considering the reduced efficacy of the antimicrobial, while the remaining 25 (71.4%) confirmed that they would stick to the usual recommended doses (Figure 1).

Another aspect targeted by the questionnaire was the criterion for selecting antimicrobials used in therapy by practicing veterinarians.

**Figure 2:**
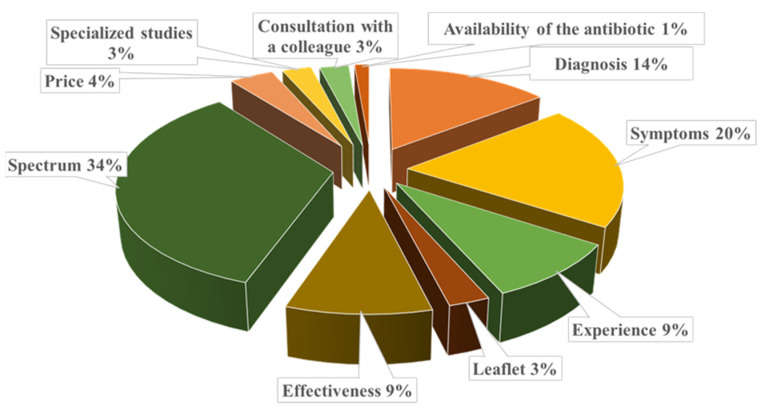
Criteria considered by Romanian veterinarians when selecting antimicrobial agents in the absence of antibiogram results. Highlighting the factors influencing their empirical antimicrobial choices when culture and sensitivity testing were not available.

The main criteria for choosing a specific antimicrobial were assessed through an open-ended question, allowing veterinarians to freely indicate the factors influencing their decision-making process. Among the surveyed veterinarians were the spectrum of action, animal symptoms, and diagnosis (Figure 2). Regarding the spectrum of action, of the 26 respondents who specified this criterion, 17 (65.3%) mentioned that they prefer a broad-spectrum antimicrobial.

The study also looked into the prophylactical use of antimicrobials. Among the veterinarians interviewed (*n* = 80), only 17 (21.3%) said they recommend or use antimicrobials for prophylactic purposes, while the remaining 63 (78.8%) responded negatively to this aspect. Additionally, of the veterinarians who stated they use antimicrobials preventively (*n* = 17, 21.3%), only seven (41%) specified that this practice is frequent for them, while the other 10 (59%) indicated that they use antimicrobials prophylactically only in rare cases.

A particularly important aspect was determining the duration of antimicrobial therapy chosen by veterinarians.

**Figure 3:**
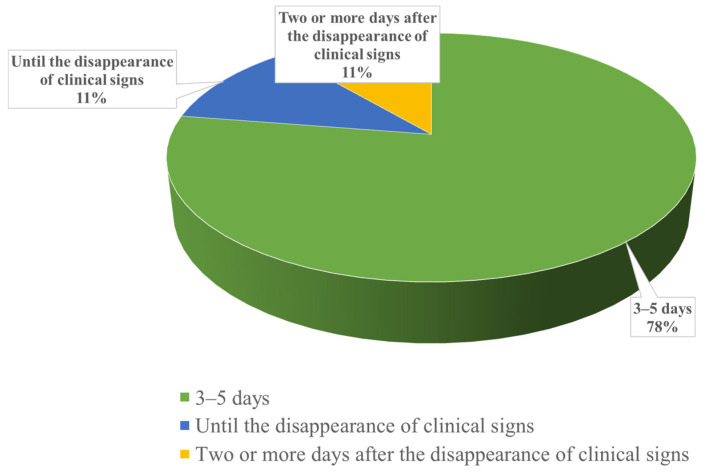
Average duration of antimicrobial therapy prescribed by Romanian veterinarians for cattle and pigs. Highlighting the typical lengths of antimicrobial treatments administered in the absence of antibiogram results.

Among the interviewed veterinarians, the majority (*n* = 62, 78%) reported administering antimicrobials for a standard duration of 3–5 days, regardless of the disappearance of clinical signs. A smaller number of veterinarians (*n* = 9, 11%) indicated that they continue therapy until the clinical signs disappear, and an equal number (*n* = 9, 11%) reported maintaining therapy for an additional two days after the disappearance of clinical signs (Figure 3).

Another aspect examined was the proportion of veterinarians who use combinations of antimicrobials. Slightly over half of the surveyed veterinarians (*n* = 48, 60%) stated that they use combinations of antimicrobials, while the rest (*n* = 32, 40%) specified that they do not use antimicrobial combinations.

**Figure 4:**
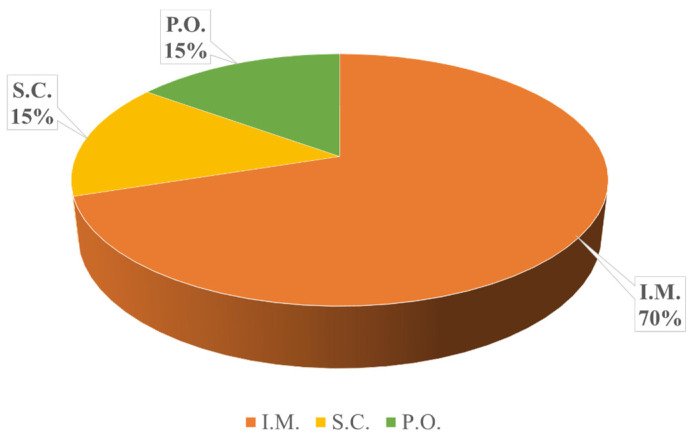
Most frequently used routes of antimicrobial administration by Romanian veterinarians in cattle and pig practice. Highlighting the predominant methods of antimicrobial delivery, such as oral, injectable, or other routes, as reported by the respondents.

Another important aspect of this questionnaire was determining the most frequently used route of antimicrobial administration. Among the 80 surveyed veterinarians, the majority (*n* = 56, 70%) prefer intramuscular administration, followed by oral administration (*n* = 12, 15%) and subcutaneous administration (*n* = 12, 15%) (Figure 4).

In antimicrobial therapy, it is crucial to adhere to the medication package insert, leaflet, and the manufacturer’s instructions. Of the veterinarians surveyed, 71 (89%) stated that they always comply with the instructions and follow the medication package insert, while only nine (11%) mentioned that they do not always adhere to the manufacturer’s instructions.

A primary step in choosing an antimicrobial is performing a patient consultation and establishing a diagnosis. Data from the questionnaires indicate that only nine veterinarians (11%) prescribe antimicrobials without first performing a consultation with the animal, while the remaining 71 (89%) stated that they have never prescribed antimicrobials without prior consultation. Regarding the response to ineffective antimicrobial treatment, among the 80 veterinarians surveyed, 78 (97%) chose to switch to a different antimicrobial, while 2 (3%) opted to increase the dosage of the initially selected antimicrobial.

### 3.3. The Antibiotics Administered in C-Sections, Abomasopexies, and Mastitis in Cattle

Surgical interventions often involve the use of antimicrobials, so veterinarians were asked about the antimicrobials they administer during C-sections in cattle and abomasopexy procedures, as well as the timing of antimicrobial administration.

C-section, abomasopexy, and mastitis were selected as key situations for evaluating antibiotic use due to their clinical relevance and the potential risk of antimicrobial resistance (AMR) development in veterinary practice. C-section is a common surgical intervention in cattle, particularly in cases of dystocia, where prophylactic and therapeutic antibiotic administration is essential to prevent postoperative infections and ensure proper wound healing. Abomasopexy also necessitates antibiotic use to mitigate the risk of peritoneal and incisional infections following surgical intervention. Mastitis, a prevalent and economically significant disease in dairy cattle, frequently requires antimicrobial treatment to control bacterial infections and prevent herd-wide outbreaks. Given the high antibiotic usage associated with these conditions, evaluating veterinarians’ prescribing patterns in such scenarios is crucial for identifying potential misuse or overuse, which could contribute to AMR development.

**Figure 5:**
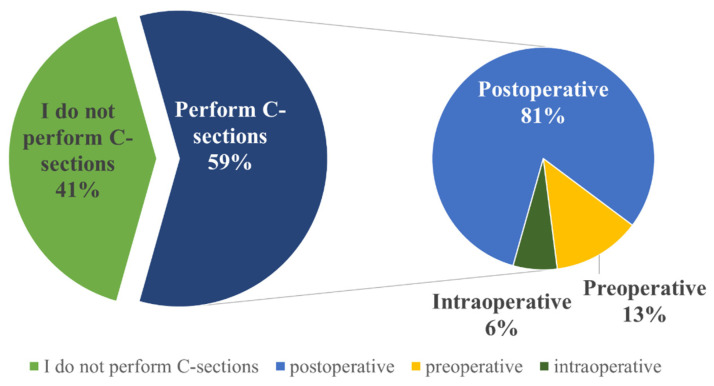
Timing of antimicrobial administration during cesarean sections in cattle as reported by Romanian veterinarians. Highlighting the timing of antimicrobial administration, preoperative, intraoperative, or postoperative during cesarean procedures.

Out of the 80 respondents, 33 (41%) do not perform C-sections. Among those who do perform this procedure (*n* = 47, 59%), the majority choose to administer antimicrobials postoperatively (*n* = 38, 81%), while a small percentage administer them preoperatively (*n* = 6, 13%) or intraoperatively (*n* = 3, 6%) (Figure 5). The most commonly administered antimicrobials are combinations of beta-lactam and aminoglycoside antimicrobials (procaine benzyl penicillin + dihydrostreptomycin sulfate) (Table 2).

**Figure 6:**
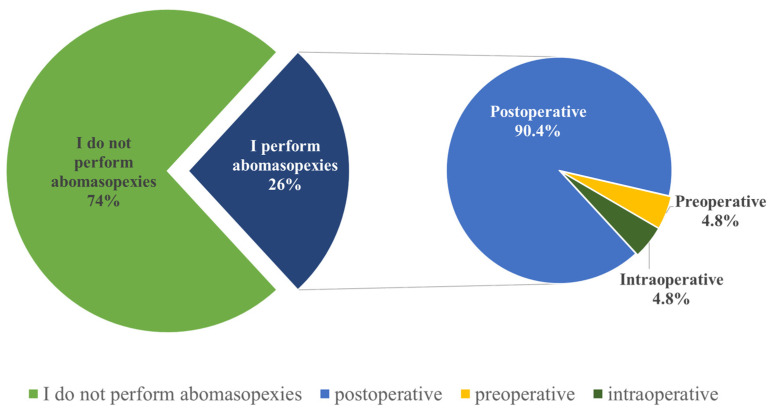
Timing of antimicrobial administration during abomasopexy procedures in cattle as reported by Romanian veterinarians. Highlighting the timing of antimicrobial administration, preoperative, intraoperative, or postoperative, during abomasopexy surgeries.

Regarding abomasopexy procedures, 59 (74%) veterinarians responded that they do not perform this surgical operation, while the remaining 21 (26%) veterinarians do perform it (Figure 6). Among these 21 veterinarians, the majority (*n* = 19, 90.4%) stated that they administer antimicrobials postoperatively, with only a small percentage administering antimicrobials preoperatively (*n* = 1, 4.8%) or intraoperatively (*n* = 1, 4.8%) (Figure 6).

Regarding the most frequently administered antimicrobials (18 respondents out of the 21), they are similar to those used in C-sections, consisting of combinations of beta-lactam and aminoglycoside antimicrobials (procaine benzyl penicillin + dihydrostreptomycin sulfate) (Table 2).

These questions aimed to identify the most commonly administered antimicrobials for mastitis, digestive disorders, reproductive system conditions, and respiratory ailments in cattle. In open-ended questions where veterinarians were asked to specify commonly used antimicrobials, most veterinarians provided an answer. However, some mentioned either the class or the product containing the antimicrobial. In cases of mastitis, antimicrobials predominantly belong to the beta-lactam class (*n* = 19, 25.3%), specifically amoxicillin (*n* = 7, 9.3%), amoxicillin + clavulanic acid (*n* = 6, 8%), penicillin (*n* = 5, 6.7%), and ampicillin (*n* = 1, 1.3%). Following these, combinations of tetracyclines, aminoglycosides, and cyclic polypeptides are frequently utilized (*n* = 17, 20.7%). This combination includes tetracycline + basic neomycin + bacitracin; constituents found in the product Mastijet (*n* = 17, 22.7%) (Table 2).

### 3.4. Antibiotics Administered in Digestive, Reproductive, and Respiratory Tract Conditions in Cattle and Pigs

In the case of digestive disorders in cattle, the surveyed veterinarians (71 respondents to this question) most commonly used combinations of antimicrobials from the beta-lactam and aminoglycoside classes (*n* = 21, 29.6%), specifically procaine benzylpenicillin and dihydrostreptomycin sulfate (*n* = 18, 25.4%) (Table 3).

Similarly, in the case of antimicrobial therapy in pigs, the focus was also on determining the most commonly administered antimicrobials for disorders of the digestive, reproductive, and respiratory tracts.

For disorders affecting the digestive tract in pigs, the surveyed veterinarians (74 respondents to this question) most commonly use antimicrobials from the fluoroquinolone class (*n* = 21, 28.4%), specifically enrofloxacin (*n* = 21, 28.4%) (Table 3).

For reproductive system disorders in cattle, the surveyed veterinarians (71 respondents to this question) most frequently utilize antimicrobials from the tetracycline class (*n* = 16, 22.5%), specifically oxytetracycline (*n* = 16, 22.5%) (Table 4).

For disorders affecting the reproductive tract in pigs, the surveyed veterinarians (70 respondents to this question) most commonly use combinations of antimicrobials from the beta-lactam and aminoglycoside classes (*n* = 21, 30%), specifically procaine benzylpenicillin + dihydrostreptomycin sulfate (*n* = 17, 24.3%) and penicillin + streptomycin (*n* = 4, 5.7%) (Table 4).

In the case of respiratory disorders in cattle, the interviewed veterinarians (76 respondents to this question) stated that they most commonly use antimicrobials from the fluoroquinolone class (*n* = 18, 23.7%), specifically enrofloxacin (*n* = 13, 17.1%) and marbofloxacin (*n* = 5, 6.6%), as well as combinations of antimicrobials from the beta-lactam and aminoglycoside classes (*n* = 18, 23.7%), specifically procaine benzylpenicillin + dihydrostreptomycin sulfate (*n* = 15, 19.7%) and penicillin + streptomycin (*n* = 3, 3.9%) (Table 5).

For disorders affecting the respiratory tract in pigs, the surveyed veterinarians (78 respondents to this question) most commonly use, similar to disorders of the reproductive tract, combinations of antimicrobials from the beta-lactam and aminoglycoside classes (*n* = 21, 26.9%), specifically procaine benzylpenicillin + dihydrostreptomycin sulfate (*n* = 16, 20.5%) and penicillin + streptomycin (*n* = 5, 6.4%) (Table 5).

### 3.5. Antibiotics Administered in the Case of Castrations in Pigs

**Figure 7:**
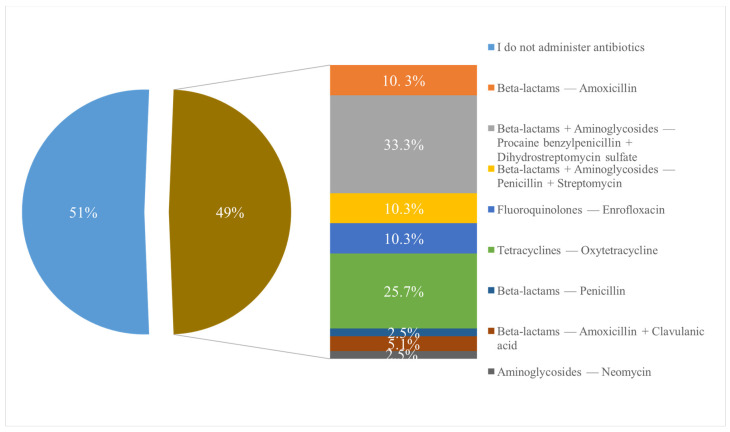
Antimicrobial agents administered following castration procedures in swine, as reported by Romanian veterinarians. Highlighting the antimicrobial agents most frequently prescribed to prevent or treat infections associated with surgical castration of male pigs.

Additionally, it was intended to determine whether veterinarians administer antimicrobials when castrating piglet and, if so, which ones they use. Among the total respondents, 41 (51%) veterinarians stated that they do not administer any antimicrobials, while 39 (49%) responded that they do administer antimicrobials, mentioning that the most frequently used combinations are from the beta-lactam and aminoglycoside classes, specifically procaine benzylpenicillin + dihydrostreptomycin sulfate (*n* = 13, 33.3%) and penicillin + streptomycin (*n* = 4, 10.3%), specifying the commercial product known as Pen-Strep and the class of tetracyclines (*n* = 10, 25.7%) (Figure 7).

### 3.6. The Level of Concern Among Veterinarians Regarding AMR and the Reasons Why Veterinarians Believe Their Colleagues Use Antibiotics Incorrectly

Another aspect addressed in this questionnaire was obtaining information on whether veterinarians with over 5 years of experience currently use the same antimicrobials as they did 5–10 years ago.

Out of the 80 veterinarians surveyed, 44 (55%) had less than 5 years of experience and could not answer this question. However, among the remaining eligible 36 veterinarians, 19 (24%) stated that they use the same antimicrobials, while 17 (21%) said that they use different antimicrobials.

**Figure 8:**
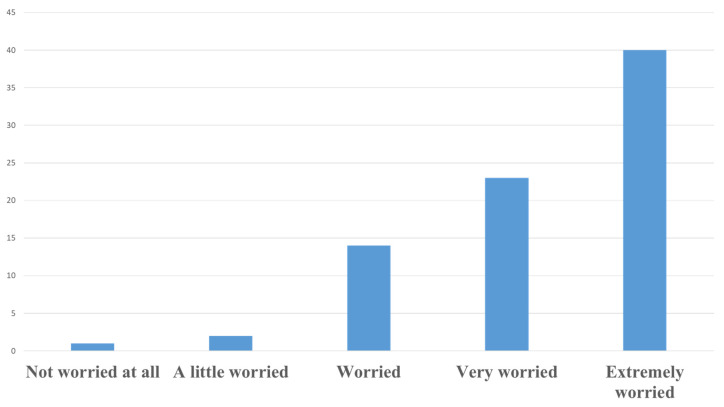
Concern levels of Romanian veterinarians regarding antimicrobial resistance (AMR). Highlighting their perceived threat levels of AMR to animal and public health. Responses were categorized as “high concern”, “moderate concern”, or “low concern”, reflecting the varying degrees of urgency attributed to AMR by the veterinary professionals surveyed.

In our study, half of the veterinarians surveyed (50%) reported being extremely concerned about the phenomenon of AMR, with an additional 28.7% indicating they were very concerned (Figure 8). When comparing responses by sex, the proportion of male veterinarians expressing extreme concern (50.85%) was only marginally higher than that of females (47.62%). This small difference suggests that there is no meaningful variation in levels of concern between male and female veterinarians. Similarly, a slightly higher proportion of female veterinarians (33.33%) were very concerned compared with males (27.12%), but this difference is also minimal and does not support the existence of a sex-based disparity in concern levels. These findings suggest that concern regarding AMR is uniformly high among veterinarians, regardless of sex. When grouping respondents into two broader age categories, those aged 25–35 years reported a lower level of extreme concern (44.82%) compared with those over 35 years (56.25%). This suggests a potential trend of increasing concern with age, although further studies with larger samples are needed to confirm this observation.

The veterinarians who participated in the survey were asked about their opinions on how their colleagues use antimicrobials. In their responses, 68% (*n* = 48) of them believed that there were errors in the antimicrobial therapy used by their colleagues.

The veterinarians’ explanations for why their colleagues make mistakes fall into the following categories: “ignorance/incompetence”, “insufficient continuing professional education”, “profit”, and “I don’t know/I don’t wish to comment”.

Here are the veterinarians’ arguments regarding the reasons why their colleagues make mistakes, categorized accordingly: 10.4% (*n* = 5) answered that they do not know or do not wish to comment; 23% (*n* = 11) considered that this happens for reasons of economic profit; 33.3% (*n* = 16) argued that the reason may be ignorance/incompetence, and 33.3% (*n* = 16) stated that this aspect is due to insufficient continuing professional training.

These insights highlight various perspectives among veterinarians regarding the reasons behind antimicrobial misuse, emphasizing the need for improved education, professional development, and awareness of AMR.

## 4. Discussion

Based on the analysis of the survey data, several key points emerge. The majority of veterinarians are young (80% are between 25 and 35 years old), and a large proportion (67.5%) have less than five years of experience, indicating a relatively young veterinary workforce. Regarding the use of antibiograms, only 50% of veterinarians use them, which could be explained by financial constraints or limited access to laboratory services.

Concerning cost influence on antimicrobial selection, 44% of veterinarians opt for cheaper alternatives, which may compromise treatment efficacy and contribute to antimicrobial resistance (AMR). Additionally, the preference for broad-spectrum antimicrobials is common, reflecting a frequent practice that, if not properly managed, could favor resistance.

Overall, these results highlight the need for improved education, better access to resources to combat AMR, and more judicious use of antibiograms.

Performing an antibiogram before administering antimicrobials allows for the selection of the most effective antimicrobial, thereby avoiding the use of antimicrobials to which pathogens have already developed resistance. In our study, half of the responding veterinarians answered negatively, and the other half responded affirmatively regarding the performance of antibiograms prior to antimicrobial administration. These results are consistent with a study conducted in Kentucky by Odoi et al. [22], which found that slightly over half of the responding veterinarians base their antimicrobial use on laboratory test results.

Findings similar to ours were obtained in a study conducted in Serbia by Vidović et al. [23], where it was found that 49% of responding veterinarians routinely use antibiograms before antimicrobial use. In contrast to our results, a study in Ireland by O’Connor et al. [24] found that a smaller percentage of veterinarians routinely use antibiograms before antimicrobial administration: only 0.9% use them daily, and 18.4% use them weekly. However, 54.4% still use antibiograms, but only when antimicrobial therapy fails.

In our study, the most common criteria veterinarians use when not performing antibiograms include spectrum (34%), symptoms (19.7%), and diagnosis (14.4%) (Figure 2). These criteria differ from those reported by veterinarians in a study conducted by Pozza et al. in Italy [25], in which the main criteria for antimicrobial selection were efficacy, field experience, withdrawal period, and scientific knowledge. Similarly, the criteria for antimicrobial selection mentioned by veterinarians in our study differ from those reported by Vijay et al. in India [26], in which the most frequent criteria were experience, antimicrobial availability, and cost.

Regarding the antimicrobial spectrum, out of the 26 respondents who mentioned it, 17 (65.3%) preferred broad-spectrum antimicrobials. A considerable number of veterinarians chose broad-spectrum antimicrobials in our study; however, prolonged use of these antimicrobials can lead to AMR [27]. In some cases, when feasible, it is more beneficial to use a narrow-spectrum antimicrobial instead of a broad-spectrum one, as the broad-spectrum antimicrobial may have detrimental effects on the normal bacterial flora [28].

The prophylactic use of antimicrobials actively contributes to and accelerates the emergence of AMR. It is crucial to eliminate such practices where the negative effects outweigh the benefits.

Prophylaxis is indicated exclusively in situations when it is allowed under EU regulations, such as when there is a high risk of infection spread in a group of animals and no viable alternatives exist. Its use is restricted to individual animals and must be justified by a veterinarian, ensuring compliance with EU strategies for reducing antimicrobial reliance. Moreover, it is only permitted for the period during which alternative disease management methods are being implemented [20].

In our study, only 21.3% of interviewed veterinarians stated that they recommend or use antimicrobials prophylactically. Similar results were obtained in a study by Saman et al. in Pakistan [29], in which 33% of responding veterinarians recommended antimicrobials prophylactically.

Regarding antimicrobial treatment, the duration should generally follow the instructions provided in the leaflet. An insufficient administration period may lead to the recurrence of the infection and simultaneously select for more resistant bacterial strains, thereby accelerating the phenomenon of AMR [20]. In cases of acute infections, for an effective treatment, it is recommended that antimicrobial administration continue for a minimum of 2 more days after the disappearance of clinical signs and the confirmation of negative microbiological tests [28]. Conversely, treatment should not be unnecessarily prolonged, as this places pressure on the body’s commensal bacterial flora and tips the competitive balance between the commensal flora and the antimicrobial-resistant pathogenic flora in favor of the resistant pathogenic strains [20]. Among the veterinarians interviewed in this study, the majority stated that they administer antimicrobials for a standard period of 3–5 days (Figure 3).

In our study, 60% of interviewed veterinarians stated they use antimicrobial combinations. Our results are similar to those found in a study by Saman et al. in Pakistan [29], in which 53% of responding veterinarians prescribed antimicrobial combinations.

In our study, 89% of interviewed veterinarians indicated that they adhere to antimicrobial product labels. Our findings align with those of Saman et al. in Pakistan [29], in which 79% of responding veterinarians followed antimicrobial product labels for dosage calculations.

The oral administration of antimicrobials has been shown to promote AMR, making it the most harmful route in this regard. Opting for alternative administration routes can reduce this effect [30].

Surgical interventions frequently involve antimicrobial use, sometimes excessively. Timing the administration optimally is crucial because administering the antimicrobial preoperatively protects the body during surgical procedures, guarding against intraoperative contamination [31]. However, in our study, a small number of veterinarians reported administering antimicrobials preoperatively for both C-sections and abomasopexy, which is consistent with the findings of other studies [31,32,33].

Regarding antimicrobials used for C-sections and abomasopexy in our study, veterinarians stated they most commonly use a combination of procaine benzyl penicillin and dihydrostreptomycin sulfate (Table 2). Our results differ from those of other studies, where veterinarians indicated that they more frequently administer procaine penicillin followed by oxytetracycline [33].

Among the most common conditions requiring antimicrobial therapy in cattle are mastitis, digestive system disorders, reproductive system disorders, and respiratory disorders [34].

Mastitis represents the primary condition for antimicrobial use in dairy cows and also has a negative economic impact on dairy farms [35]. In our study, antimicrobials most commonly used for mastitis include a combination of tetracycline HCL, basic neomycin, and bacitracin, which are found in the commercial product Mastijet, as mentioned by responding veterinarians (Table 2). Our results differ from those of a study conducted in Serbia by Vidović et al. [23], in which responding veterinarians reported using enrofloxacin most frequently for mastitis, and from those of a study in Australia by Tree et al. [36], in which veterinarians reported using cloxacillin most frequently.

In the current study, veterinarians commonly used beta-lactam and aminoglycoside combinations for digestive disorders in cattle, whereas De Briyne et al. [37] reported polymyxins as the most frequently used antimicrobials for diarrhea in cows and calves.

For digestive tract disorders in pigs, fluoroquinolones were the most reported antimicrobials in our study, differing from De Briyne et al. [37], where polymyxins were preferred for colibacillosis and dysentery.

As De Briyne’s study dates back to 2014, the observed decline in polymyxin (colistin) use may reflect the impact of extensive campaigns discouraging their use, a positive development in antimicrobial stewardship.

In our study, veterinarians reported using oxytetracycline most frequently for reproductive system disorders in cattle (Table 4). Our results differ from those of a study by Webb et al. [34], where ceftiofur was most frequently mentioned for metritis treatment.

Regarding respiratory disorders in cattle, veterinarians in our study most frequently mentioned using a combination of procaine benzylpenicillin and dihydrostreptomycin sulfate (Table 5). Our findings differ from those of a study by Webb et al. [34], where florfenicol was most frequently used for pneumonia in calves and heifers, and from those of a study by De Briyne et al. [37], where macrolides were mentioned most frequently for respiratory disorders in cattle.

In cases of respiratory tract disorders in pigs, the veterinarians in our study most frequently mentioned combinations of beta-lactam and aminoglycoside antimicrobials (Table 5). Our findings differ from those of a study by De Briyne et al. [37], where tetracyclines were most frequently mentioned for respiratory disorders in pigs.

Regarding the veterinarians’ reasoning behind their colleagues’ errors, the majority attributed them to “ignorance/incompetence” and “insufficient professional training”. These categories likely stem from a lack of knowledge, consistent with the findings of a study by Llanos-Soto et al. [38], where veterinarians mainly cited lack of knowledge as a primary reason.

A study from Serbia highlights the role of targeted AMS education in improving veterinary students’ understanding of antimicrobial resistance (AMR) and responsible antimicrobial use. The findings show that structured training enhances awareness of resistance mechanisms and reinforces the importance of stewardship practices. While short-term improvements were evident, the long-term impact on clinical decision-making remains to be explored. Given the growing threat of AMR, integrating comprehensive AMS education into veterinary curricula is essential to shaping future veterinarians’ approach to antimicrobial use and resistance mitigation [39].

In a study from Cyprus, veterinarians and food-producing animal operators demonstrated varying levels of knowledge, attitudes, and practices regarding antimicrobial resistance (AMR) and antimicrobial use (AMU). While veterinarians exhibited sufficient knowledge, notable gaps remained, particularly in restricting the use of priority antimicrobials and broad-spectrum antibacterials. The continued prescription of Category B antibiotics highlights the need for stricter regulatory enforcement and targeted education. Additionally, the lack of a significant association between knowledge levels and prescribing practices suggests that external factors, such as economic pressures or client expectations, may influence decision-making. Among operators, positive attitudes were strong predictors of responsible antimicrobial practices, emphasizing the importance of awareness campaigns. Strengthening collaboration between veterinarians and animal producers, alongside comprehensive governance and policy enforcement, is essential to mitigating AMR in the Cypriot veterinary sector [40].

In a study from the UK, consumer perceptions of antimicrobial resistance (AMR) and its association with antibiotic use in food animals were explored. The results revealed a significant lack of understanding, with around 50% of respondents indicating uncertainty by answering “don’t know” to many questions. While 40% agreed that antibiotics for treating diseases in farm animals provide more benefits than harm, the high proportion (44%) of undecided respondents reflects a widespread uncertainty about AMR. This suggests that public awareness about the long-term consequences of antibiotic overuse in agriculture remains limited, highlighting the urgent need for more comprehensive education on the dangers of AMR and its potential impact on human health [41].

In a study conducted in seven cities in Southern and Central China, university students’ knowledge, attitudes, and practices regarding antibiotic use in humans and food-producing animals were assessed. The results revealed significant gaps in understanding, with less than half of the students answering knowledge-related questions correctly. Only a small percentage, ranging from 21.47% to 29.98%, demonstrated a proper understanding of basic antibiotic concepts and their use in both humans and animals. Additionally, only 21.49% and 28.50% of students paid attention to antibiotic content in food from animals or avoided purchasing food with antibiotics, respectively. Factors influencing better knowledge and practices included being male, older, having a medical background, studying at prestigious universities, and having a higher family income. These findings highlight the need for targeted educational interventions to address the knowledge gaps and improve antibiotic-related practices among students, emphasizing the risks of antibiotic resistance and providing guidance on how to mitigate it [42].

The discrepancies between the findings of this study and similar studies may stem from several factors. Differences in the demographics of the surveyed veterinarians, such as age and experience, could influence their antimicrobial practices, with younger veterinarians possibly adhering more closely to emerging guidelines. Geographic factors, including regional variations in antimicrobial resistance (AMR) patterns and regulatory enforcement, may also play a role. Additionally, discrepancies could arise from differences in veterinary education and training, particularly in antimicrobial stewardship (AMS), which can vary across countries. Economic factors, such as the cost of antimicrobials and access to diagnostic resources like antibiograms, may also contribute to differing practices. These factors highlight the importance of considering local contexts when interpreting the results and developing strategies to improve antimicrobial use and resistance mitigation.

### 4.1. Limitations

This study has several limitations, including the absence of a detailed statistical analysis of the data. Additionally, the small sample size and the vagueness of some respondents’ answers may have affected the accuracy and generalizability of the findings.

Further limitations include potential biases inherent to this type of survey. Sampling bias may have influenced the representativeness of the responses, while self-selection bias, due to the online distribution method, could have led to the overrepresentation of certain perspectives. Incomplete responses and the reliance on self-reported data without external verification may have further impacted data reliability. Additionally, open-ended questions carry the risk of misinterpretation, which could affect the consistency of the responses. The study was also subject to constraints such as a limited time frame and limitations in questionnaire design, which may have restricted the depth of insights obtained.

### 4.2. Strengths

The study has several strengths, including addressing a critical public health issue, AMR, and a clear objective—evaluating the use of antimicrobials by veterinarians in Romania. The research provides valuable insight into the level of awareness and persistent errors in antimicrobial administration.

## 5. Conclusions

The study highlights notable differences between Romanian cattle and pig veterinarians in their use and opinions regarding antibiotics. While many veterinarians demonstrate good prescribing practices, there remains room for improvement in antibiotic stewardship. Our findings do not provide evidence that concern over antimicrobial resistance is increasing, as this aspect was not explicitly measured. Additionally, the extent to which veterinary professionals are exposed to information on the risks of antimicrobial resistance was not explored in this study.

The results indicate that while there are gaps in knowledge and practice, a considerable number of veterinarians adhere to appropriate prescribing principles. These findings emphasize the need for continued awareness and adherence to best practices to ensure responsible antibiotic use in veterinary medicine.

## Figures and Tables

**Table 1:** Descriptive data of 80 Romanian cattle and pig veterinarians who completed a questionnaire on their antimicrobial practices in 2022.

**Age**				
N = 80 %	25–35 years	36–45 years	46–65 years	>65 years
	64 80	5 6.25	10 12.5	1 1.25
**Gender**				
N = 80 %	Female		Male	
	21 21.3		59 73.8	
**Professional Experience**				
N = 80 %	0–5 years	5–10 years	10–20 years	≥20 years
	54 67.5	8 10	7 8.8	11 13.7

**Table 2:** Antimicrobial agents commonly used by Romanian veterinarians for cesarean sections, abomasopexy procedures, and mastitis treatment in cattle. Highlighting the antimicrobial agents most frequently prescribed for cesarean sections, abomasopexy procedures, and mastitis treatment in cattle. (The order of antimicrobials in the table is according to the most utilized class.)

Disease	Antimicrobial Class	Total Number of Answers (%)	Antimicrobial	Total Number of Answers (%)
C-section (47)	C C D D D D B	25 (53.2%) 17 (36.2%) 4 (8.5%) 1 (2.1%)	Procaine benzyl penicillin + dihydrostreptomycin sulfate (Pen-Strep, Cen-a-pen, Combi-kel) Penicillin + Streptomycin Penicillin Amoxicillin Amoxicillin + clavulanic acid (Synulox) Oxytetracycline Ceftiofur	19 (40.4%) 6 (12.8%) 7 (14.9%) 5 (10.6%) 5 (10.6%) 4 (8.5%) 1 (2.1%)
Abomasopexy (18)	C C D D B B D C	7 (33.3%) 4 (19%) 3 (14.3%) 2 (9.5%) 1 (4.8%) 1 (4.8%)	Procaine benzyl penicillin + dihydrostreptomycin sulfate (Pen-Strep, Combi Kel) Penicillin + Streptomycin Amoxicillin + clavulanic acid (Synulox) Penicillin Ceftiofur Enrofloxacin Oxytetracycline Gentamycin	4 (19%) 3 (14.3%) 3 (14.3%) 1 (4.8%) 3 (14.3%) 2 (9.5%) 1 (4.8%) 1 (4.8%)
Mastitis	D D D D B C C B B B C C C C D D C D	19 (25.3%) 17 (22.7%) 8 (10.7%) 8 (10.7%) 8 (10.7%) 5 (6.7%) 4 (5.3%) 4 (5.3%) 1 (1.3%)	Amoxicillin Amoxicillin + clavulanic acid (Synulox) Penicillin Ampicillin Tetracycline HCL + Basal neomycin + Bacitracin (Mastijet) Procaine benzyl penicillin + dihydrostreptomycin sulfate (Pen-Strep, Combi Kel) Penicillin + Streptomycin Marbofloxacin (Marbox, Marboxil) Enrofloxacin Cefquinome Cefalexin Gentamycin Kanamycin Streptomycin Tetracycline Tylosin Erythromycin Sodium novobiocin + neomycin sulfate + procaine benzylpenicillin + dihydrostreptomycin sulfate (Gamaret)	7 (9.3%) 6 (8%) 5 (6.7%) 1 (1.3%) 17 (22.7%) 7 (9.3%) 1 (1.3%) 4 (5.3%) 4 (5.3%) 6 (8%) 2 (2.7%) 3 (4%) 1 (1.3%) 1 (1.3%) 4 (5.3%) 3 (4%) 1 (1.3%) 1 (1.3%)

Legend: Category B (Restrict): Highest priority antimicrobials for human medicine; veterinary use should be restricted (e.g., third- and fourth-generation cephalosporins). Category C (Caution): Alternatives exist in human medicine; use in animals should be limited to cases where Category D substances are ineffective. Category D (Prudence): First-line antimicrobials for veterinary use; recommended with standard prudence.

**Table 3:** Most frequently administered antimicrobials for the treatment of digestive disorders in cattle and pigs. Highlighting the antimicrobial agents most commonly used to treat enteric diseases such as colibacillosis, scours, and dysentery. The table includes information on the antimicrobial class, specific compounds, and their typical routes of administration (oral, injectable, or in-feed), as well as their pharmacokinetic properties.

Species	Class of Antimicrobials	Number of Answers (%)	Utilized Antimicrobials	Number of Answers (%)
Cattle	C	21 (29.6%)	Procaine benzyl penicillin + dihydrostreptomycin sulfate (Pen-Strep, Combi Kel)	18 (25.4%)
	C		Penicillin + Streptomycin	3 (4.2%)
	B	19 (26.8%)	Enrofloxacin	19 (26.8%)
	D	13 (18.3%)	Oxytetracycline	10 (14.1%)
	D		Tetracycline	3 (4.2%)
	D	9 (12.7%)	Amoxicillin	4 (5.6%)
	D		Amoxicillin + clavulanic acid	2 (2.8%)
	D		Penicillin	3 (4.2%)
	C	4 (5.6%)	Gentamicin	2 (2.8%)
	C		Neomycin	1 (1.4%)
	C		Streptomycin	1 (1.4%)
	C	3 (4.2%)	Lincomycin + Spectinomycin	3 (4.2%)
	C	1 (1.4%)	Spectinomycin (Spectam)	1 (1.4%)
	C	1 (1.4%)	Lincomycin	1 (1.4%)
Pigs	B	21 (28.4%)	Enrofloxacin	21 (28.4%)
	C	15 (20.3%)	Procaine benzyl penicillin + dihydrostreptomycin sulfate	14 (18.9%)
	C		Penicillin + Streptomycin	1 (1.4%)
	D	10 (13.5%)	Oxytetracycline	10 (13.5%)
	C	8 (10.8%)	Streptomycin	5 (6.8%)
	C		Gentamicin	3 (4.1%)
	D	8 (10.8%)	Amoxicillin (Amoxicrid, Betamox)	4 (5.4%)
	D		Amoxicillin + clavulanic acid	3 (4.1%)
	D		Penicillin	1 (1.4%)
	C	4 (5.4%)	Lincomycin + Spectinomycin	4 (5.4%)
	C	4 (5.4%)	Spectinomycin (Spectam)	4 (5.4%)
	C	2 (2.7%)	Tylosin	2 (2.7%)
	C	1 (1.4%)	Lincomycin (Lincomix)	1 (1.4%)
	C	1 (1.4%)	Tiamulin	1 (1.4%)

Legend: Category B (Restrict): Highest priority antimicrobials for human medicine; veterinary use should be restricted (e.g., third- and fourth-generation cephalosporins). Category C (Caution): Alternatives exist in human medicine; use in animals should be limited to cases where Category D substances are ineffective. Category D (Prudence): First-line antimicrobials for veterinary use; recommended with standard prudence.

**Table 4:** Antimicrobial agents commonly used by Romanian veterinarians for treating reproductive system diseases in cattle and pigs. Highlighting the antimicrobial agents most frequently prescribed for reproductive conditions such as mastitis, endometritis, and metritis in cattle, as well as reproductive tract infections in pigs.

Species	Class of Antimicrobials	Number of Answers (%)	Utilized Antimicrobials	Number of Answers (%)
Cattle	D	16 (22.5%)	Oxytetracycline	16 (22.5%)
	C	14 (18.7%)	Procaine benzyl penicillin + dihydrostreptomycin sulfate (Pen-Strep)	13 (18.3%)
	C		Penicillin + Streptomycin	1 (1.4%)
	C	11 (15.5%)	Gentamicin	9 (12.7%)
	C		Streptomycin	1 (1.4%)
	C		Kanamycin	1 (1.4%)
	D	11 (15.5%)	Amoxicillin (Clamoxyl)	6 (8.5%)
	D		Amoxicillin + clavulanic acid	1 (1.4%)
	D		Penicillin	4 (5.6%)
	B	10 (14.1%)	Ceftiofur	6 (8.5%)
	B		Cefapirin (Metricure)	4 (5.6%)
	B	8 (11.3%)	Enrofloxacin	6 (8.5%)
	B		Marbofloxacin	2 (2.8%)
	C	1 (1.4%)	Spectinomycin (Spectam)	1 (1.4%)
Pigs	C	21 (30%)	Procaine benzyl penicillin + dihydrostreptomycin sulfate	17 (24.3%)
	C		Penicillin + Streptomycin	4 (5.7%)
	D	16 (22.9%)	Oxytetracycline	16 (22.9%)
	D	14 (20%)	Amoxicillin	8 (11.4%)
	D		Penicillin	5 (7.1%)
	D		Amoxicillin + clavulanic acid	1 (1.4%)
	B	10 (14.3%)	Enrofloxacin	7 (10%)
	B		Marbofloxacin	3 (4.3%)
	C	9 (12.9%)	Gentamicin	7 (10%)
	C		Streptomycin	2 (2.9%)

Legend: Category B (Restrict): Highest priority antimicrobials for human medicine; veterinary use should be restricted (e.g., third- and fourth-generation cephalosporins). Category C (Caution): Alternatives exist in human medicine; use in animals should be limited to cases where Category D substances are ineffective. Category D (Prudence): First-line antimicrobials for veterinary use; recommended with standard prudence.

**Table 5:** Antimicrobial agents commonly used by Romanian veterinarians for treating respiratory diseases in cattle and pigs. Highlighting the antimicrobial agents most frequently prescribed for respiratory conditions such as pneumonia, pleuropneumonia, and other respiratory tract infections in cattle and pigs.

Species	Class of Antimicrobials	Number of Answers (%)	Utilized Antimicrobials	Number of Answers (%)
Cattle	B	18 (23.7%)	Enrofloxacin (Baytril)	13 (17.1%)
	B		Marbofloxacin (Marboxil, Marbox)	5 (6.6%)
	C	18 (23.7%)	Procaine benzyl penicillin + dihydrostreptomycin sulfate ()	15 (19.7%)
	C		Penicillin + Streptomycin	3 (3.9%)
	D	14 (18.4%)	Amoxicillin	8 (10.5%)
	D		Amoxicillin + clavulanic acid	1 (1.3%)
	D		Penicillin	5 (6.6%)
	C	10 (13.2%)	Tulathromycin	6 (7.9%)
	C		Tylosin	3 (3.9%)
	C		Spiramycin	1 (1.3%)
	C	4 (5.3%)	Spectinomycin	4 (5.3%)
	B	4 (5.3%)	Ceftiofur	4 (5.3%)
	C	3 (3.9%)	Florfenicol	3 (3.9%)
	D	3 (3.9%)	Oxytetracycline	3 (3.9%)
	C	2 (2.6%)	Sulfadoxine + Trimethoprim (Borgal)	2 (2.6%)
Pigs	C	21 (26.9%)	Procaine benzyl penicillin + dihydrostreptomycin sulfate	16 (20.5%)
	C		Penicillin + Streptomycin	5 (6.4%)
	D	14 (17.9%)	Amoxicillin	8 (10.3%)
	D		Amoxicillin + clavulanic acid	2 (2.6%)
	D		Penicillin	4 (5.1%)
	B	13 (16.7%)	Enrofloxacin	10 (12.8%)
	B		Marbofloxacin	3 (3.8%)
	C	8 (10.3%)	Tylosin	4 (5.1%)
	C		Tulathromycin	3 (3.8%)
	C		Tildipirosin	1 (1.3%)
	C	7 (9%)	Florfenicol	7 (9%)
	C	4 (5.1%)	Lincomycin + Spectinomycin	4 (5.1%)
	D	3 (3.8%)	Oxytetracycline	3 (3.8%)
	C	3 (3.8%)	Streptomycin	2 (2.6%)
	C		Gentamicin	1 (1.3%)
	B	2 (2.6%)	Ceftiofur	2 (2.6%)
	C	2 (2.6%)	Spectinomycin	2 (2.6%)
	C	1 (1.3%)	Tiamulin	1 (1.3%)

Legend: Category B (Restrict): Highest priority antimicrobials for human medicine; veterinary use should be restricted (e.g., third- and fourth-generation cephalosporins). Category C (Caution): Alternatives exist in human medicine; use in animals should be limited to cases where Category D substances are ineffective. Category D (Prudence): First-line antimicrobials for veterinary use; recommended with standard prudence.

## Data Availability

The original contributions presented in this study are included in the article. Further inquiries can be directed to the corresponding authors.

## Supplementary Materials

The following supporting information can be downloaded at: https://www.mdpi.com/article/10.3390/vetsci12050408/s1. The questionnaire that was completed by the 80 veterinary doctors.
