# Supplementary material for: Questionnaire-Based Survey Regarding the Rational Usage of Antimicrobial Agents in Food-Producing Animals in Romania
Source: Vet Sci. 2025 Apr 26;12(5):408. doi: 10.3390/vetsci12050408 (PMC12115682; doi:10.3390/vetsci12050408)
Supplement: Supplementary file 1 [file vetsci-12-00408-s001.zip › vetsci- 3559485 - supplementary/vetsci-3559485-ethic.pdf]

**The Bioethics Commission**

*UNIVERSITY OF LIFE SCIENCES " King Mihai I" from Timișoara, 119, Calea Aradului, 300645, Timișoara, RO* ▪ *tel/fax: +40 256 277140*

**The Scientific Pro-Rectorate**
No. 531/08.05.2025

# To whom it may concern

*The Bioethics Commission from University Of Life Sciences " King Mihai I" from Timișoara, analyzed and come to the conclusion that the studies from the research intended to be published: Questionnaire-bazed survey regarding the rational usage of antimicrobial agents in food-producing animals in Romania from the Faculty of Veterinary Medicine Timișoara, by Ionela Popa, Kalman Imre, Adriana Morar, Ionica Iancu, Vlad Iorgoni, Timea Bochiș, Călin Pop, Alexandru Gligor, Tiana Florea, Sebastian Popa, Viorel Herman, Ileana Nichita does not need the approval of the Bioethics Commison as the reaserch does not imply the use of live animals in an experimental setting.*

*We believe that the results can be published and complies with the recent regulations on protection of animals used for experimental and other scientific purposes.*

***Head of***
***Bioethics Commission of ULS Timișoara***

***Lecturer LĂZĂRESCU CRISTIAN, DVM, PhD***



CamScanner
