# Supplementary material for: Questionnaire-Based Survey Regarding the Rational Usage of Antimicrobial Agents in Food-Producing Animals in Romania
Source: Vet Sci. 2025 Apr 26;12(5):408. doi: 10.3390/vetsci12050408 (PMC12115682; doi:10.3390/vetsci12050408)
Supplement: Supplementary file 1 [file vetsci-12-00408-s001.zip › vetsci- 3559485 - supplementary/vetsci-3559485-the survey questions.pdf]

1. Age
2. Gender

F M

3. Professional experience
   0-5 years
   5-10 years
   10-20 years
   Over 20 years
4. Do you request the performance of an antibiogram before administering an antibiotic?
   Yes No
5. If the most effective antibiotic indicated by the antibiogram has a high cost, do you choose/recommend a more affordable alternative with lower efficacy?
   Yes No
6. If you choose a less effective option because it is more affordable, do you resort to administering higher doses of the antibiotic than usual?
   Yes No
7. If you do not perform the antibiogram, what is the criterion you use to choose the antibiotic?
8. Do you use/recommend the administration of antibiotics for prophylactic purposes?
   Yes No
   If yes, how often?
9. What is the average duration of antibiotic treatment?
   3-5 days
   Until the disappearance of clinical signs
   Two or more days after the disappearance of clinical signs
10. Do you also use antibiotic combinations?
    Yes No
11. What is the most common route of antibiotic administration?
12. Do you always administer antibiotics according to the protocol/package insert?

    Yes No

13. Have you ever prescribed antibiotics without examining the animal?

    Yes No

14. If the antibiotic therapy does not yield the expected results:
    Change the antibiotic
    Increase the usual dose
15. When do you use antibiotics in the case of a cesarean section in cattle?
    Preoperative
    Intraoperative
    Postoperative
    I do not perform cesarean sections
16. What is the antibiotic used in the case of a cesarean section in cattle?
17. When do you use antibiotics in the case of abomasopexy in cattle?
    Preoperative
    Intraoperative
    Postoperative
    I do not perform abomasopexy

18. What is the antibiotic used in the case of abomasopexy in cattle?
19. Which antibiotic do you most often use in the case of mastitis in cattle?
20. What is the antibiotic you usually use in the case of respiratory diseases in cattle?
21. What is the antibiotic you usually use in the case of digestive diseases in cattle?
22. What is the antibiotic you usually use in the case of reproductive system diseases in cattle?
23. What is the antibiotic you usually use in the case of respiratory diseases in pigs?
24. What is the antibiotic you usually use in the case of digestive diseases in pigs?
25. What is the antibiotic you usually use in the case of reproductive system diseases in pigs?
26. Do you administer antibiotics in the case of castrations in pigs?

    Yes No

    If yes, what antibiotic do you resort to?
27. Are the antibiotics you use most frequently now the same as the ones you used 5-10 years ago?

    Yes No I have less than 5 years of experience
28. How concerned are you about the phenomenon of antimicrobial resistance?

    1. Not at all concerned - 5. Very concerned
29. Do you think some colleagues use antibiotics improperly?

    Yes No

    If yes, what do you think the reasons might be?
